# Sprinting performance of individuals with unilateral transfemoral amputation: compensation strategies for lower limb coordination

**DOI:** 10.1098/rsos.221198

**Published:** 2023-03-08

**Authors:** Mingyu Hu, Toshiki Kobayashi, Genki Hisano, Hiroto Murata, Daisuke Ichimura, Hiroaki Hobara

**Affiliations:** ^1^ Department of Biomedical Engineering, Faculty of Engineering, The Hong Kong Polytechnic University, Hong Kong, People's Republic of China; ^2^ Department of Systems and Control Engineering, Tokyo Institute of Technology, Tokyo 152-8550, Japan; ^3^ Research Fellow of Japan Society for the Promotion of Science (JSPS), Tokyo 102-0083, Japan; ^4^ Artificial Intelligence Research Center, National Institute of Advanced Industrial Science and Technology, Tokyo 135-0014, Japan; ^5^ Department of Mechanical Engineering, Tokyo University of Science, Chiba 278-8510, Japan; ^6^ Faculty of Advanced Engineering, Tokyo University of Science, Tokyo 125-8585, Japan

**Keywords:** continuous relative phase, gait, prosthetic, run, running-specific prosthesis

## Abstract

Understanding the sprinting patterns of individuals with unilateral transfemoral amputation (uTFA) is important for designing improved running-specific prostheses and for prosthetic gait rehabilitation. Continuous relative phase (CRP) analysis acquires clues from movement kinematics and obtains insights into the sprinting coordination of individuals with uTFA. Seven individuals with uTFA sprinted on a 40 m runway. The spatio-temporal parameters, joint and segment angles of the lower limbs were obtained, and CRP analysis was performed on thigh-shank and shank-foot couplings. Subsequently, the asymmetry ratios of the parameters were calculated. Statistical analyses were performed between the lower limbs. Significant differences in the stance time, stance phase percentage, ankle joint angles and CRP of the shank-foot coupling (*p* < 0.05) were observed between the lower limbs. The primary contributor to these differences could be the structural differences between the lower limbs. Despite the presence of different coordination features in the stance and swing phases between the lower limbs, no significant difference in the coordination patterns of the thigh-shank coupling was observed. This may be a compensation strategy for achieving coordination patterns with improved symmetry between the lower limbs. The results of this study provide novel insights into the sprinting movement patterns of individuals with uTFA.

## 1. Introduction

Understanding the gait patterns of individuals with amputation is crucial for developing improved running-specific prostheses and for prosthetic gait rehabilitation [1–3]. For prosthesis design, running-specific prostheses could be optimized based on the real sprinting prosthetic gait profile. For prosthetic gait rehabilitation, the sprinting gait profile obtained using a prosthesis could aid in designing a target standard for new individuals with amputation to train their sprinting or running as part of their gait training. In individuals with amputation, the ability to sprint is restored to a limited extent using running-specific prostheses [4]. However, biological differences due to limb loss between individuals with and without amputation still exist and contribute to gait differences [5]. For sprinting movement, individuals with transfemoral amputation employed compensatory strategies to coordinate their functionally and anatomically asymmetric limbs. The intact limb often has larger peak extensor and flexor moments than the prosthetic limb in the hip and knee joints [6]. Because the prosthetic limb cannot efficiently generate forces against the ground during the push-off phase, the intact limb must compensate by generating a larger force in the stance phase during running [7]. These forces and moments cause changes in the kinematics (i.e. joint/segment angles and angular velocities). Distinctive features between the lower limbs of individuals with transfemoral amputation exist in the kinematics during running. The thigh segment exhibited a decreased range of motion, and the knee joint was overextended for a longer period in the prosthetic limb than in the intact limb during the stance phase. Furthermore, compared to the intact limb, the time required by the prosthetic limb to reach maximum dorsiflexion in the ankle joint was longer [8]. In individuals with transfemoral amputation that wore the Terry Fox jogging prostheses during jogging, the knee joint on the prosthetic limb was hyperextended during the entire stance phase [9]. Functional asymmetries were also suggested to be a part of compensatory strategies. For instance, the ankle joint on the intact side often exhibited premature midstance plantar flexion to assist the toe clearance of the prosthetic limb during the single-limb support phase [10].

Movement patterns arise from the organization of the neuromuscular system and involve multiple joints and segments. Investigating the isolated joint and segments might not effectively reflect the interaction effects or coordination of the musculoskeletal system because the compensatory mechanisms are often spread among several body parts [7]. The coordination of multiple body parts to generate movement patterns supports the theory that movement patterns are produced by controlling multiple degrees of freedom in the human body [11]. Movement coordination in kinematics can be quantified by dynamic system theory using a continuous relative phase (CRP) method [11,12]. The status of the system is based on energy exchange. When applying this method to gait analysis, the segments of the lower limb are traditionally modelled as a pendulum [13,14]. In this manner, the lower limb segments oscillate in a quasi-sinusoidal portrait or a limit cycle shape during cyclic movements, which can be a dynamic system. In addition, the system has constant energy exchange during the gait cycle. When new behaviours are generated by the neuromuscular system, the status of the system changes, and energy imbalances arise. The overall state of the system can be described by the current status and changing rate [11]. Consequently, the angles and angular velocities at a certain moment can effectively reflect the phase space state (phase angle) of a lower limb segment. The coordination between two lower limbs can be quantified using the differences in the phase space states (relative phase angle) between two lower limb segments. The CRP angle can reflect the coordination changes over a period of time. In the CRP method, the mean absolute relative phase (MARP) and the deviation phase (DP) represent the coordination pattern and the variability of the CRP used at a specific time or phase of the gait cycle, respectively. A high (low) MARP indicates an out-of-phase (in-phase) relationship, and an excessively high or low DP represents coordination instability in the coupling segments [12]. Specifically, an excessively low DP indicates a rigid movement pattern and restricted adaptability, while an excessively high DP represents an unstable movement pattern.

To improve the running performance of individuals with unilateral transfemoral amputation (uTFA), running-specific prostheses are widely used. Coordinating asymmetries between lower limbs during sprinting is challenging for individuals with amputations [15]. However, to the best of our knowledge, research on the sprinting coordination of individuals with uTFA is lacking, and only one study, which aimed at modelling the lower limb based on joints, has analysed the walking coordination of individuals with uTFA [16]. Although the results of this study did not include MARP values, they indicated that the prosthetic limb exhibited a significantly larger DP in knee-ankle coupling during the stance phase and this relationship reversed during the swing phase. Therefore, coordination patterns in kinematics and asymmetries between the lower limbs wearing running-specific prostheses remain unknown. Understanding coupling effects could help manufacturers optimize the coupling effects of their prostheses, as the aim of a prosthesis is to restore function to the greatest extent. An improved understanding of running-specific prosthesis sprinting coordination could maximize athletic performance through design and optimized gait training. The coordination patterns of experienced prosthesis users would help guide prosthetic gait training and rehabilitation for individuals after amputation. Specifically, minimizing the differences between experienced and new prosthesis users in CRP curves could provide valuable data for the optimization of prosthesis design.

Therefore, this study aimed to understand the coordination patterns of lower limbs in individuals with uTFA during sprinting to improve running-specific prosthesis design and prosthetic gait rehabilitation. Following the typical approach, we modelled the lower limb segments in the pendulum and incorporated the concept of dynamic system theory [11]. We focused on three segments of the lower limb, namely, the thigh, shank and foot, and investigated the CRP in the two couplings (thigh-shank and shank-foot). To compensate for the functional asymmetries in individuals with uTFA, we assumed that the coordination patterns between the lower limbs are different during sprinting. The prosthetic limb might demonstrate larger variability in coordination patterns (excessively high DP) than the intact limb, as the neuromuscular system connections of the intact limb are unimpaired. Based on this concept, we propose two hypotheses. First, in thigh-shank coupling, the prosthetic limb should exhibit a larger DP than the intact limb. Second, in shank-foot coupling, the prosthetic limb might demonstrate a more significant in-phase relationship during the swing phase than the intact limb, since the prosthetic limb is a blade with limited degrees of freedom. To test the hypotheses, the coordination curve, asymmetry ratios of basic gait parameters, and coordination parameters (MARP and DP) of the lower limbs were compared.

## 2. Materials and methods

### 2.1. Participants

Seven individuals with uTFA participated in the study (table 1). Participants were categorized as functional levels K3-K4 [17], with an age of 32.7 ± 11.2 y, height of 1.63 ± 0.09 m, mass of 56.5 ± 7.9 kg, time since amputation of 11.9 ± 8.6 y and sprinting speed of 5.85 ± 0.45 m s^−1^. The inclusion criteria of the individuals with uTFA included no neuromuscular disorders, no lower limb functional limitations in either limb, and regular exercise or training for at least 1 year before the experiment. This study was approved by the local ethics committee, and all procedures followed the Declaration of Helsinki (1983). Participants were informed of the test content, and their consent was obtained.

**Table 1.** Demographic data. Abbreviations: F: female; M: male; TF; transfemoral; KD; knee-disarticulation; s.d.: standard deviation.

participant	gender (M/F)	age (y)	height (m)	mass (kg)	amputated side	etiology	time since amputation (y)	residual limb length	foot strike types (intact limb)	prosthetic knee	prosthetic foot	running speed (m s^−1^)
1	M	42	1.67	57.2	left	cancer	6	long TF	rearfoot	3S80	Sprinter 1E90 (cat.2)	5.68
2	M	52	1.70	66.6	left	trauma	29	KD	forefoot	3S80	Sprinter 1E90 (cat.3)	6.02
3	F	38	1.49	43.9	right	infection	15	long TF	forefoot	3S80	Sprinter 1E90 (cat.2)	5.63
4	M	21	1.67	56.4	left	cancer	18	short TF	forefoot	3S80	Sprinter 1E90 (cat.3)	5.34
5	F	32	1.56	47.4	right	trauma	6.5	middle TF	forefoot	3S80	Sprinter 1E90 (cat.2)	5.59
6	F	18	1.56	58.3	right	trauma	3.5	middle TF	forefoot	3S80	Sprinter 1E90 (cat.2)	5.89
7	M	26	1.75	66.0	right	trauma	5.2	middle TF	forefoot	3S80	Sprinter 1E90 (cat.3)	6.83
mean	–	32.7	1.63	56.5	–	–	11.9	–	–	–	–	5.85
s.d.	–	11.2	0.09	7.9	–	–	8.6	–	–	–	–	0.45

### 2.2. Experimental procedures

Before the experiment, participants performed warm-up exercises to familiarize themselves with the experimental environment. All participants performed static and dynamic stretching and several types of locomotion, including walking, skipping, hopping and jogging, for more than 15 min. Thereafter, each participant performed the maximum sprinting trials. Subsequently, they performed maximal sprinting on a 40 m indoor straight runway. Seven 60 cm × 40 cm force plates (five BP400600-1000 and two BP400600-2000, AMTI, Watertown, MA, USA) were set on the ground. A motion capture system (VICON MX system, Oxford Metrics Ltd., UK) with 20 cameras and an effective capture distance of approximately 22 m from the starting line was used. The results from each trial were collected for further analysis. Only trials in which the participant stepped within the force platform boundaries were counted as successful. There were two to three successful trials for each leg. Participants were allowed adequate rest intervals between trials.

### 2.3. Data collection and analysis

**Figure 1.**
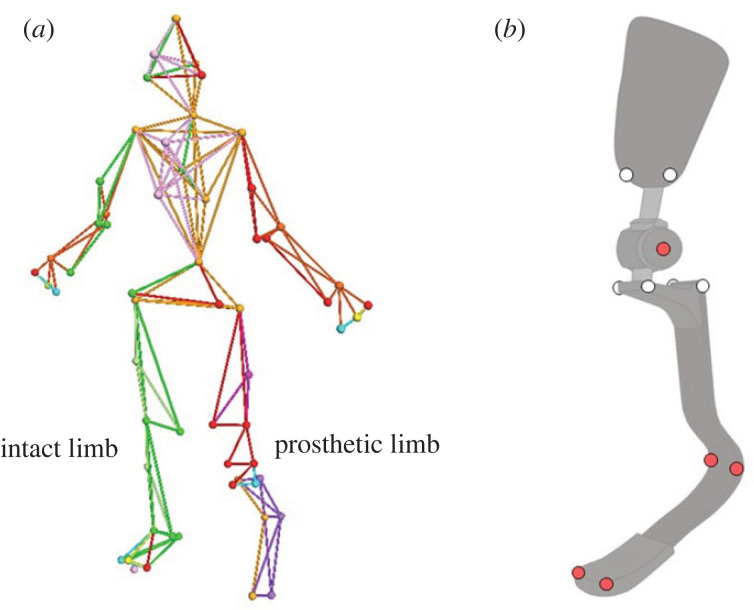
Lower limb model definition (*a*) and retroreflective marker locations on the transfemoral prosthesis (*b*). The red marks indicate the markers used for kinematics analyses, while white ones were not.

Before the experiment, 58 retroreflective markers were attached to the body of each participant (figure 1). Markers on the prosthetic limb, including the prosthetic shank and foot segments, were located based on a previous study [6]. In this model, the prosthetic ‘ankle’ joint was represented by the most acute point on the midline between the medial and lateral edges of the prosthesis curvature. The remaining markers were attached following the Helen Hayes marker set [18].

The gait cycle was normalized to 101 points. The GRF data and three-dimensional marker positions were collected at sampling frequencies of 2000 and 200 Hz, respectively. A fourth-order zero-lag Butterworth filter was applied to the raw GRF data at a cut-off frequency of 75 Hz and raw three-dimensional marker positions at a cut-off frequency of 20 Hz. The GRF data were used to define gait events by setting a threshold value of 40 N [19]. For steps not contacting the force plates, gait events were identified using a kinematic algorithm depending on the limbs. In the intact limb, the identification of the initial contact was based on the peak vertical acceleration of the first metatarsal and heel markers for the forefoot and rearfoot strike types, respectively [20]. The toe-off was identified based on the peak vertical acceleration of the first metatarsal marker for both foot strike patterns [21]. In the prosthetic limb, the peak vertical acceleration of the most distal marker attached to the prosthetic foot was used because the two peaks in vertical acceleration could coincide with the initial contact and toe-off in this limb. The three-dimensional coordinate data were used to calculate the sagittal plane joint angles (ankle, knee and hip) for basic joint kinematics and sagittal plane segment angles (thigh, shank and foot) for further coordination analysis.

The Hilbert transform method was applied for CRP analysis [12]. The segment angles (θ) of each participant were normalized (equation (2.1)) and considered as a time series signal (equation (2.2)), where *i* and *t* represent the *i*th and *t*th points in the analytical data, respectively. A custom-made MATLAB script was used to conduct the Hilbert transform of the signal in the previous step (equations (2.3) and (2.4)), where *j* represents the imaginary part. The ‘unwarp’ function in MATLAB was applied to calculate the phase angle (equation (2.5)). The results of the arctan function ranged between ±90° by definition; however, the phase angle of a polarized coordinate must have a full range of 360°. The unwarp function ensured that the final phase angle would retain the previous value and not skip to the ±90° range. The CRP angles of the thigh-shank and shank-foot couplings were calculated by subtracting the proximal angle from the distal one (equation 2.6). CRP angles with ranges outside ±180° were adjusted to ±180° by adding or subtracting 360°. The MARP and DP of each gait cycle were calculated based on the CRP values obtained in the last step (equations (2.7) and (2.8)). MARP evaluates the coordination pattern, and DP represents the variability of the overall coordination at a specific time or phase of the gait cycle. A MARP (or CRP) value of 0° indicates that the coupling performs an in-phase coordination pattern, while an increasing distance from 0° indicates an out-of-phase relationship in the coupling [12].

2.1 $$\bar{\theta }=\theta -\min(\theta )-\left(\frac{\max(\theta )-\min(\theta )}{2}\right),$$

2.2 $$\theta (t)={\bar{\theta }}_{\left(i\right)},\;i=1,2,\ldots ,n\;,$$

2.3 $$H(t)\;=H(\theta (t))=\;\theta (t)*\frac{1}{\pi t},$$

2.4 ζ(t)=θ(t)+jH(t),

2.5 $$\emptyset_{rp}=\arctan\left[\frac{H(t_{i})}{\theta (t_{i})}\;\right],\;i=1,2,\ldots ,n$$

2.6 $$\emptyset_{\mathrm{CRP}}=\emptyset_{rp-\mathrm{distal}}-\;\emptyset_{rp-\mathrm{proximal}},$$

2.7 $$\mathrm{MARP}=\overset{n}{\underset{i=1}{\sum }}\frac{|\emptyset_{i\;th-\mathrm{crp}}|}{n},\;i=1,2,\ldots ,n$$

2.8 $$\mathrm{and}\;DP=\frac{\sum_{i=1}^{n}|SD_{i}|}{n},i=1,2,\ldots ,n.$$

The CRP values of the thigh-shank and shank-foot couplings were plotted as CRP curves. The time of the local minimum or maximum of the percentage of the gait cycle was recognized in each trial and averaged for the lower limbs. Because a positive slope implies that the distal angle leads the proximal one and vice versa, the local minimum or maximum values might indicate moments of changes in the coordination patterns [12].

Subsequently, the asymmetry ratio for each gait parameter was calculated [22]. For individuals with uTFA, the asymmetry ratio was the ratio of the gait parameter of the prosthetic limb to that of the intact limb. An asymmetry ratio of 1 indicated a symmetrical relationship between the gait parameters for the two lower limbs. An asymmetry ratio greater than 1 indicated that the gait parameter was larger for the prosthetic limb than for the intact limb, while an asymmetry ratio between 0 and 1 indicated that the gait parameter was smaller for the prosthetic limb than for the intact limb. In this study, the asymmetry ratios of the spatio-temporal, kinematic and MARP parameters were investigated.

### 2.4. Statistical analysis

The Shapiro–Wilk test was conducted to verify data normality. For each gait parameter with a normal distribution, a paired *t*-test was performed to investigate differences between the lower limbs. For gait parameters without a normal distribution, the Wilcoxon signed-rank test was used to analyse the differences between the limbs. The statistical analyses were performed using SPSS (IBM SPSS Statistics 22, SPSS Inc., Chicago, IL) with a significance level of *p* < 0.05. Subsequently, the significance of the paired *t*-test and Wilcoxon signed-rank test were adjusted by false discovery rate correction. The effect size was calculated using Cohen's d and Cliff's delta methods for the normal and non-normal distributed parameters, respectively.

For the CRP curves (thigh-shank and shank-foot), SPM (statistical parametric mapping) was then applied to investigate the significance between the prosthetic and intact limbs [23]. The significance level in the SPM analysis was adjusted to 0.025 according to Bonferroni's correction.

## 3. Results

The demographic data of the seven participants are listed in table 1. The mean running speed in this study was 5.85 ± 0.45 m s^−1^.

### 3.1. Spatio-temporal and kinematic parameters

As listed in table 2, no significant differences in step length were observed between the lower limbs of individuals with uTFA (*p* = 0.24). However, the stance time and stance phase percentage were significantly lower for the intact limb than for the prosthetic limb, each with *p* = 0.01.

**Table 2.** Mean value, normality, *p*-value and asymmetry ratio of the parameters. Abbreviations: Y: Yes; N: No; Ext: extension; Flex: flexion; D-Flex: dorsiflexion; P-Flex: plantarflexion; RoM: range of motion; MARP thigh-shank: mean absolute relative phase of thigh-shank coupling; MARP shank-foot: mean absolute relative phase of shank-foot coupling; DP thigh-shank: deviation phase of thigh-shank coupling; DP shank-foot: deviation phase of shank-foot coupling; TP1 thigh-shank: time (per cent of gait cycle) to the first peak (P1) of thigh-shank coupling CRP curve in figure 2*d*; TP1 shank-foot: time (per cent of gait cycle) to the first peak (P1) of shank-foot coupling CRP curve in figure 2*e*; TP2 shank-foot: time (per cent of gait cycle) to the second peak (P2) of shank-foot coupling CRP curve in figure 2*e*.

parameters	intact limb	prosthetic limb	normality	*p*-adjusted	effect size	asymmetry ratio
step length (m)	1.57 ± 0.06	1.46 ± 0.19	N	0.24	0.63	0.93 ± 0.10
stance time (s)	0.12 ± 0.01	0.13 ± 0.01	Y	0.01 ^a^	0.72	1.14 ± 0.09
stance phase (%)	22.33 ± 2.23	26.01 ± 1.73	Y	0.01 ^a^	0.65	1.17 ± 0.10
peak ankle D-Flex (°)	11.39 ± 3.70	13.35 ± 1.28	Y	0.36	0.25	1.33 ± 0.49
peak ankle P-Flex (°)	−40.98 ± 8.95	−4.08 ± 1.28	Y	<0.01 ^a^	2.04	0.11 ± 0.05
ankle RoM (°)	52.37 ± 7.25	17.43 ± 1.04	Y	<0.01 ^a^	2.39	0.34 ± 0.03
peak knee Flex (°)	127.28 ± 8.96	97.37 ± 4.62	Y	<0.01 ^a^	2.06	0.77 ± 0.06
peak knee Ext (°)	–	−5.41 ± 2.35	–	–	–	–
knee RoM (°)	118.27 ± 7.34	102.78 ± 3.86	Y	0.01 ^a^	0.93	0.87 ± 0.07
peak hip Flex (°)	73.39 ± 6.16	54.00 ± 12.75	Y	0.01 ^a^	0.31	0.73 ± 0.15
peak hip Ext (°)	−24.41 ± 6.89	−22.54 ± 4.26	N	0.61	0.68	0.99 ± 0.29
hip RoM (°)	97.80 ± 4.04	76.54 ± 10.31	Y	0.01 ^a^	0.96	0.78 ± 0.10
MARP thigh-shank (°)	61.07 ± 6.03	55.55 ± 9.61	Y	0.38	0.24	0.92 ± 0.22
MARP shank-foot (°)	12.73 ± 1.44	5.15 ± 0.67	Y	<0.01 ^a^	2.39	0.41 ± 0.08
DP thigh-shank (°)	1.97 ± 0.75	1.51 ± 0.58	Y	0.38	0.24	–
DP shank-foot (°)	1.26 ± 0.41	0.79 ± 0.40	Y	0.15	0.41	–
TP1 thigh-shank (%)	27.86 ± 1.81	35.00 ± 2.88	Y	0.01 ^a^	1.05	–
TP1 shank-foot (%)	5.43 ± 1.68	6.00 ± 1.31	N	0.53	0.20	–
TP2 shank-foot (%)	27.71 ± 1.16	25.57 ± 1.99	Y	0.07	0.47	–

^a^indicates a statistical significance level at *p* < 0.05.

**Figure 2.**
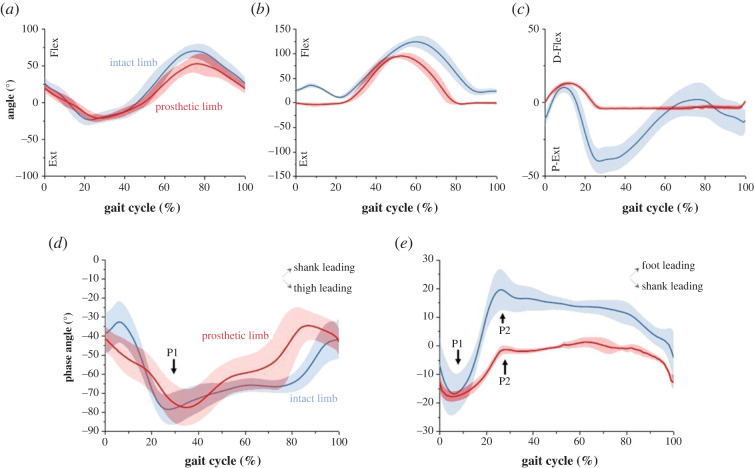
Results of joint angles and CRP curves. The red and blue colours represent the prosthetic limb and intact limb, respectively. (*a–c*) Results for hip, knee and ankle joints. In these three plots, D-Flex: dorsiflexion; P-Flex: plantarflexion; RoM: range of motion; Ext: extension. (*d*) CRP curve of the thigh-shank coupling, with the positive and negative slopes representing shank-leading and thigh-leading patterns, respectively. P1 in this (*d*) is the first common peak at which both lower limbs change from a thigh-leading to a shank-leading pattern. (*e*) CRP curve of the shank-foot coupling, with the positive and negative slopes representing foot-leading and shank-leading patterns, respectively. P1 in this (*e*) is the first common peak at which both lower limbs change from a shank-leading to a foot-leading pattern. P2 in this (*e*) for the intact limb is the second peak at which the intact limb changes from a foot-leading to a shank-leading pattern. P2 for the prosthetic limb is the second peak at which the prosthetic limb changes from a foot-leading pattern to an in-phase relationship.

Table 2 and figure 2(*a–c*, respectively) illustrate the kinematics of the ankle, knee and hip joints. There were no significant differences in peak ankle dorsiflexion, hip flexion and extension between prosthetic and intact limbs. The ankle joint showed a significantly higher peak of plantarflexion and range of motion in the intact limb than in the prosthetic limb (*p* < 0.01). For the knee joint, significant differences were observed between the lower limbs. The intact limb only performed knee flexion throughout the gait cycle, while the prosthetic limb had an extension of −5.41 ± 2.35°. There were significantly higher peaks of flexion and range of motion in the knee on the intact limb than on the prosthetic limb (*p* < 0.01 for knee flexion, *p* = 0.01 for range of motion). The peak flexion and range of motion of the hip joint were significantly higher for the intact limb than for the prosthetic limb (*p* = 0.01).

For the spatio-temporal parameters, the step length on the intact limb was larger than that on the prosthetic limb, and the stance time and stance phase duration were longer on the prosthetic limb than the intact limb (asymmetry ratios of 0.93 for step length, 1.14 for stance time and 1.17 for stance phase). For the kinematics, the maximum ankle plantarflexion and range of motion were close to zero while the maximum ankle dorsiflexion was greater than one and was the largest value among all asymmetry ratios (asymmetry ratios of 1.33 for maximum ankle dorsiflexion, 0.34 for ankle range of motion and 0.11 for ankle plantarflexion). The knee and hip joint parameters were all larger for the intact limb (0.87 for knee range of motion, 0.73 for maximum hip flexion and 0.78 for hip range of motion), even though the maximum hip extension was very close to symmetrical (0.99 for maximum hip extension).

### 3.2. Coordination

Among the coordination measures, only the MARP of the shank-foot on the intact limb demonstrated a significantly greater out-of-phase pattern than that of the prosthetic limb (*p* < 0.01). There were no significant differences between the intact and prosthetic lower limbs in the MARP of the thigh-shank coupling or the DP of either thigh-shank or shank-foot coupling. The asymmetry ratios of the MARP of thigh-shank and shank-foot couplings were 0.92 ± 0.22 and 0.41 ± 0.08, respectively.

The CRP curves are depicted in figure 2*d,e*. Positive values indicate that the distal segment is ahead of the proximal segment in the phase space and vice versa. A positive (negative) slope indicates that the distal (proximal) one is leading the other [11,12]. In the thigh-shank coupling, the shank was always ahead of the thigh in the phase space in both limbs as shown by the negative values throughout the entire gait cycle. In the prosthetic limb, the thigh was leading the shank until approximately 35% (P1) and then back to the thigh-leading pattern at the end of the gait (table 2). The intact limb demonstrated a significantly earlier change from thigh-leading pattern to shank-leading pattern than the prosthetic limb (TP1thigh-shank, Intact: prosthetic = 27.86 ± 1.81: 35.00 ± 2.88, *p* = 0.01). Two other clear features are shown in this plot. First, the intact limb was observed with a shank-leading pattern between 0% to 10% of the gait cycle, whereas the prosthetic limb had the thigh leading during the entire stance phase. Second, a shank-leading pattern was observed on the intact limb throughout the swing phase, whereas the prosthetic limb maintained a shank-leading pattern for approximately 80% of the gait cycle and then demonstrated a thigh-leading pattern.

In the shank-foot coupling, as shown in figure 2*e*, a transition of shank-leading pattern to foot-leading pattern at around 6% of the gait cycle from the initial contact was observed in both lower limbs, with no significant difference found in the changing time (table 2, TP1shank-foot, intact: prosthetic = 5.43 ± 1.68: 6.00 ± 1.31, *p* = 0.53). The intact limb ended the foot-leading pattern later than the prosthetic limb, but this is not significant (table 2, TP2shank-foot, intact: prosthetic = 27.71 ± 1.16: 25.57 ± 1.99, *p* = 0.07). The intact limb then retained a shank-leading pattern until the end of the gait cycle, whereas the prosthetic limb fluctuated around 0° until the end of the swing phase, and then demonstrated a shank-leading pattern to the end of the gait cycle.

**Figure 3.**
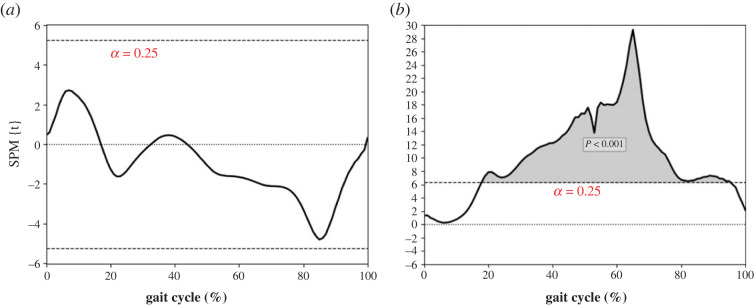
SPM results of CRP curves. (*a*) CRP thigh-shank and (*b*) CRP shank-foot curves. A longer dashed line indicates the threshold for *α* level = 0.025. Grey-shaded parts indicate significant differences between the lower limbs.

The SPM analysis results are shown in figure 3. The CRP curves in thigh-shank coupling failed to reach the significant level (figure 3*a*). In the shank-foot coupling, significant differences are observed from 20% to almost the end of the gait cycle (figure 3*b*, *p* < 0.001).

## 4. Discussion

This study investigated the coordination of the lower limbs of individuals with uTFA during sprinting. We proposed two hypotheses, namely, in thigh-shank coupling, the prosthetic limb should exhibit a larger DP than the intact limb, and in shank-foot coupling, the prosthetic limb might demonstrate a more in-phase relationship during swing phase than the intact limb, since the prosthetic limb is a blade with limited degrees of freedom. Hypothesis I was not supported as thigh-shank coupling demonstrated both similar coordination patterns and variability of the coordination throughout the gait cycle, as evidenced by the similar MARP and DP in thigh-shank couplings. Hypothesis II was supported since the MARP in shank-foot coupling on the prosthetic limb demonstrated a significant in-phase coordination pattern during the gait cycle, and the SPM plot also revealed significant differences between the two lower limbs from the swing phase.

During sprinting, individuals with uTFA demonstrated significant differences in the spatio-temporal and kinematic parameters between the lower limbs. In the spatio-temporal parameters, the prosthetic limb showed a longer stance time and phase than the intact limb. This finding is in line with a previous study on individuals with transtibial amputation [24]. A possible reason is the economic force generation strategy [21]. A longer stance time on the prosthetic limb might be a compensation strategy, with the prosthetic limb requiring a longer stance time to generate an equivalent vertical ground reaction impulse and compensate for weaker force generation [7]. Concerning joint kinematics, a significantly limited range of motion was found in the joints on the prosthetic limb than in the intact limb. In the ankle joint, dorsiflexion between the lower limbs was similar, and the limited range of motion of the prosthetic limb was primarily attributable to reduced peak ankle plantarflexion. This was also supported by the strong asymmetry in ankle plantarflexion (asymmetry ratio of 0.11 for ankle plantarflexion). The prosthetic ankle was unable to plantarflex and modulate the same range of motion as the intact ankle because of the lack of ankle plantarflexors [25]. In the knee joint, the limited range of motion of the prosthetic limb was mainly due to the peak knee flexion angle. Although the residual limb has muscles, these muscles (mainly hamstrings) may not be strong enough to control prosthetic knee flexion [26]. Some degree of prosthetic knee flexion in the swing phase might be achieved by the forward movement of the residual thigh and backward swing inertia of the prosthetic shank. However, the flexion of the prosthetic limb matched that of the intact limb because the intact limb knee flexion is dominated by active muscles. The hip joint demonstrated a limited range of motion on the prosthetic limb identical to walking [27]. The smaller hip range of motion might be due to smaller hip moments and muscles in the prosthetic limb than in the intact limb. Another possible reason might be mechanical constraints due to pelvis-socket interference [28].

Symmetrical coordination was achieved by individuals with uTFA, as thigh-shank coupling can maintain the MARP at a symmetrical level (0.92 for thigh-shank MARP asymmetry ratio) between the prosthetic and intact limbs. The SPM revealed the same results as MARP (thigh-shank) between the lower limbs during the gait cycle as no significant differences were found during the whole gait cycle (figure 3*a*). There was no significant difference in the MARP (thigh-shank), DP (thigh-shank) and DP (shank-foot), meaning that the thigh-shank parts of both limbs had a similar out-of-phase relationship during the entire gait cycle [11]. In the shank-foot coupling, the prosthetic limb performed more in-phase coordination than the intact limb. This means that the shank-foot coupling in the prosthetic limb is more synchronized than that in the intact limb, and the two segments are moving in synchrony [12]. This result accords with common sense because the shank-foot part of the prosthetic limb is a blade. Thus, the shank and foot segments of the prosthetic limb are naturally more synchronized than those of the intact limb. In terms of coordination variability, the similar DP between the lower limbs suggested both limbs could maintain the variabilities of coordination patterns at a similar level during the gait cycle.

The compensatory strategies might be suggested through the coordination patterns in the two lower limbs. Individuals with transfemoral amputation achieved symmetrical coordination (symmetrical MARP) with asymmetrical spatio-temporal and kinematic parameters. Despite the consistency in the coordination patterns of the lower limbs in thigh-shank coupling (no significance in MARP thigh-shank), there were different coordination features between the prosthetic limb and intact limb during the stance and swing phases (figure 2*d*). For the thigh-shank coupling, the intact limb started with a shank-leading pattern until 10% of the gait cycle, followed by a thigh-leading pattern during the stance phase. Meanwhile, the prosthetic limb adopted a thigh-leading pattern throughout the entire stance phase. This difference might be the compensatory strategy that the prosthetic limb cannot actively generate force in the shank part, and thus the coupling can only use the thigh-leading pattern during the stance phase. During the swing phase, the intact limb maintained a shank-leading pattern until the end of the gait cycle. However, the prosthetic limb showed a shank-leading pattern, then switched to a thigh-leading pattern at approximately 80% of the gait cycle and maintained this to the end of the cycle. The prosthetic knee reached full extension earlier than the biological knee, at 80% of the gait cycle (figure 2*b*) and this may explain the reversion to a thigh-leading pattern in the prosthetic limb. At this point in the gait cycle, there is no space for the shank to extend the knee angle; hence, the thigh segment compensates to complete the swing phase by moving and leading the shank segment to the original position. Consequently, individuals with amputation maintained symmetrical coordination in thigh-shank coupling between the lower limbs with different coordination features in the stance and swing phases.

The more in-phase coordination pattern on the prosthetic limb in MARP (shank-foot) suggested different coordination patterns in the shank-foot coupling. This might suggest that the compensation in the shank-foot coupling is not effective enough to reach symmetrical coordination patterns. For the CRP curve of shank-foot coupling, the same feature (shank leading to foot leading at 6% of the gait cycle) was observed during the stance phase and swing phase. Both limbs demonstrated a shank-leading pattern at the beginning of the gait cycle, which was then converted to a foot-leading pattern in the middle of the stance phase. During the swing phase, the prosthetic limb generally demonstrated in-phase coordination (around 0 phase angle), while the intact limb demonstrated a shank-leading pattern and returned to the original phase position. The in-phase relationship in the prosthetic limb during the swing phase was supported by the fact that the shank and foot segments of the prosthetic limb are combined as a single solid blade which will not flex when it is not in contact with the ground.

This study has several limitations. First, the causes of amputation and the residual limb length of the participants were not the same. These differences might be confounding factors. For example, compared with the traumatic type of amputation, the vascular type was suggested to result in slower walking speed and shorter stride length [29]. The femoral length also influences gait parameters, such as the shorter residual limb length reportedly being associated with a larger femoral shaft angle [30]. Second, this study had a limited number of participants due to the sprinting protocol, as it is extremely difficult to find individuals with uTFA that have proficient sprinting skills. Third, participants only performed the maximum speed sprinting, so including a range of speeds might enhance our understanding of how the coordination changes in uTFA to cope with variations. For example, a previous study revealed that from slow to fast running speeds the difference in ground reaction forces and spatio-temporal parameters demonstrated changes between lower limbs in uTFA [7]. Fourth, this study only investigated kinematic data using CRP analysis in the sagittal plane. Further studies should include other planes (transverse or coronal planes) to deepen the understanding of coordination in the sprinting of individuals with uTFA. For example, the balance during running is associated with the arm swing, and the shoulder rotation in the transverse plane influences the arm swing in the sagittal plane [31]. The combination of CRP and kinetics or electromyography might also illuminate the control strategies in individuals with amputation as the force and muscle activities are also important in generating movement patterns. Finally, only one type of running-specific prosthetic component (Knee: 3S80; Foot: Sprinter 1E90) was included in this study, but there are other knee joints and generally two common types (C-shape and J-shape) of carbon fibre prosthetic feet for runners [32]. Further investigation into other types of prosthesis could enhance our understanding of sprinting coordination in uTFA.

The findings of this study should provide a novel insight into the coordination patterns for prosthesis design and prosthetic gait rehabilitation. There are biological and functional asymmetries between lower limbs in individuals with uTFA. It is not reasonable to completely match the prosthetic limb to the intact limb since the conventional prosthetic limb cannot actively generate forces. However, recently some rehabilitation exoskeletons and powered prostheses have overcome the lack of force generation in prosthetic limbs [33,34]. While there are more in-phase coordination patterns on the prosthetic limb than the intact limb in the shank-foot couplings, designing the powered prostheses to minimize the coordination pattern differences might provide a better solution to restore limb functions in individuals with uTFA. This has the same application in prosthetic gait rehabilitation. Evaluating the segment coordination provides additional information, such as movement coordination control ability for prosthesis users.

## 5. Conclusion

In summary, individuals with uTFA may sprint by coordinating their intact and prosthetic limbs using compensatory strategies in thigh-shank coupling during the stance and swing phases to achieve similar coordination patterns. To compensate for the lack of force generation in the prosthetic shank, the prosthetic thigh-shank adopted a thigh-leading pattern throughout the whole stance phase, while the intact limb had an exchange from shank leading to thigh leading during the stance phase. During the swing phase, the prosthetic limb applied the same exchange as the intact limb performed during the stance phase, while the intact limb maintained shank leading throughout the whole swing phase. These different coordination features compensated for asymmetries to achieve symmetrical coordination. In shank-foot coupling, the prosthetic limb demonstrated a more in-phase coordination pattern, as the prosthetic ankle joint was absent in this type of prosthetic limb. The findings of this study provide novel insights into the design of prostheses and prosthetic gait rehabilitation in terms of lower limb movement coordination control.

## Data Availability

The datasets supporting this article have been uploaded as part of the electronic supplementary material [35].
